# Current health status and its risk factors of the Tsarang villagers living at high altitude in the Mustang district of Nepal

**DOI:** 10.1186/s40101-018-0181-y

**Published:** 2018-08-29

**Authors:** Sweta Koirala, Masayuki Nakano, Hiroaki Arima, Shouhei Takeuchi, Tomo Ichikawa, Takayuki Nishimura, Hiromu Ito, Basu Dev Pandey, Kishor Pandey, Takayuki Wada, Taro Yamamoto

**Affiliations:** 1 Department of International Health, Institute of Tropical Medicine, Nagasaki University, 1-12-4 Sakamoto, Nagasaki, 852-8523 Japan; 2 Leading Program, Graduate School of Biomedical Sciences, Nagasaki University, 1-12-4 Sakamoto, Nagasaki, 852-8523 Japan; 3 Graduate School of Biomedical Sciences, Nagasaki University, 1-12-4 Sakamoto, Nagasaki, 852-8523 Japan; 4 Department of Bacteriology, Institute of Tropical Medicine, Nagasaki University, 1-12-4 Sakamoto, Nagasaki, 852-8523 Japan; 5 Department of Nutrition Science, Faculty of Nursing and Nutrition, University of Nagasaki, 1-1-1 Manabino, Nagayo, Nishisonogi, Nagasaki, 851-2195 Japan; 6 Department of Society and Regional Culture, Okinawa International University, 2-6-1 Ginowan, Ginowan City, Okinawa 901-2701 Japan; 7 Department of Public Health, Graduate School of Biomedical Sciences, Nagasaki University, 1-12-4 Sakamoto, Nagasaki, Japan; 8 Department of General System Studies, Graduate School of Arts and Sciences, University of Tokyo, 3-8-1 Komaba, Meguro, Tokyo, 153-8902 Japan; 9 Everest International Clinic and Research Center, GPO 9045, Kathmandu, Nepal; 10 National Center for AIDS & STD Control, Ministry of Health and Population, GPO 9045, Teku, Kathmandu, Nepal; 11 Department of Environmental Sciences, Zoology, University of Basel, Vesalgasse 1, CH-4051 Basel, Switzerland; 12 Nepal Academy of Science and Technology, GPO 3323, Khumaltar, Lalitpur, Nepal; 13 School of Tropical Medicine and Global Health, Nagasaki University, 1-12-4 Sakamoto, Nagasaki, 852-8523 Japan

**Keywords:** High altitude, Health status, Noncommunicable diseases, Cross-sectional studies, Risk factors

## Abstract

**Background:**

Epidemiology of noncommunicable diseases (NCDs) such as obesity and diabetes mellitus (DM) are influenced by multiple hosts and environmental factors. This study aims to investigate the prevalence of NCDs and determine their risk factors among the adults residing in an isolated village situated at a rural highland of Nepal.

**Methods:**

A cross-sectional survey was conducted in a village located at 3570 m. Each 188 randomly selected participants of age ≥ 18 years old answered a questionnaire and took a full physical exam that included biomedical measurements of glycosylated hemoglobin (HbA1c).

**Results:**

The prevalence of intermediate hyperglycemia and DM was 31.6% and 4.6% respectively, and the prevalence of hypoxemia (SpO_2_ < 90%) was 27.1%. A multiple logistic regression analysis for factors for the prevalence of glucose intolerance (HbA1c ≥ 6%) revealed older age (odds ratio [OR] 1.11, 95% confidence interval [CI] 1.06–1.16, for every 1 year increase) and SpO_2_ (OR for hypoxemia 3.58, 95% CI 1.20–10.68, vs SpO_2_ ≥ 90%).

**Conclusions:**

Tibetan highlanders in the remote mountainous Mustang valley of Nepal have high prevalence of impaired glucose metabolism which could be related to hypoxemia imposed by the hypoxic conditions of high altitude living.

## Background

Low- and middle-income countries are facing with burdens of two major public health concerns, communicable and noncommunicable diseases (NCDs). While the number of patients with communicable diseases is gradually falling throughout the world, NCDs including cardiovascular diseases, cancers, and diabetes are growing rapidly and are expected to further expand throughout the world [1, 2]. Source of death in the low-income countries will predominantly be the result of NCDs in near future [3]. NCDs like diabetes mellitus (DM), obesity, and hypertension (HT) are one of the most serious public health concerns resulting in severe healthcare burdens throughout the world [1, 4]. Each year, NCDs account for 40 million deaths globally, and 80% of the premature deaths due to NCDs occur in low- and middle-income countries [5].

In Nepal, a World Bank defined low-income country, a nationwide survey on NCDs revealed that multifactorial elements were involved in the growing burden of NCDs as an emerging public health concern [6]. The World Health Organization (WHO) have reported that the proportion of deaths related to NCDs has risen from 51% in 2010 to 60% in 2014, indicating that NCDs are one of the most important health issues [4, 7]. Because of the increasing trend in NCDs, the Nepalese government established the multi-sectorial action plan for the prevention and control of NCDs [8]. In order to achieve this plan, it requires understanding about current health status including the prevalence of NCDs and their risk factors of the population at the national level. Unfortunately, Nepalese population-based studies on incidence and prevalence of NCDs and their risk factors are limited [9, 10].

Nepal has a large geographical diversity from lowland in the south to over 8000 m in mountainous areas in the north. Almost 2 million people or 6.7% of the population of Nepal live permanently in mountainous area [11]. Living at high altitudes involves long-term adaptation to harsh environment and altitude induced hypoxia. Residents living at altitude must deal with severe environmental and physiological stress and may be more likely to exhibit adaptive responses such as energy production, ventilation, and oxygen diffusion in blood stream [12]. Tsarang village (3570 m above sea level) is in an economically impoverished, remote mountainous region in Mustang district neighboring the Tibetan area of China and had long been isolated from other parts of Nepal. The restricted sacred kingdom of Mustang commenced tourism only in 1992. Because of its history, it is the most preserved region of the world and belongs to the national conservation area. Locals are adapted to the harsh environmental conditions; the economy is agro-pastoralism, and Tsarang village in Nepal has Tibetan population. However, it is facing recent globalization with access to road and trekking tourism industry. Previous studies have noted that high altitude has effects on the development NCDs including obesity and HT, suggesting that the prevalence of NCDs and risk factors for development of diseases at high altitude might be different from lowland areas [13–15]. Prevalence of obesity, specifically central obesity, is common among Tibetans. Sherpa et al. states that while there is a significant decrease in body mass index (BMI) with increasing level of altitude, there is no corresponding decrease in waist circumference (WC), suggesting that people living at high altitude have a higher prevalence of central obesity as compared to generalized total body fat measured by BMI [16]. Research shows obesity and being overweight occurred less in mountainous areas (9%) than in hills (26%) or nationwide (21%) in Nepal [6].

In this study, to understand current health condition including the prevalence of NCDs such as DM, HT, and overweight of Tsarang residents, we established a small health clinic where we carried out medical check-ups and administered a questionnaire to examine the relationship among health, lifestyle, and socioeconomic status. Our research aims were to investigate the general health condition of the Tsarang residents to determine the risk factors for the most prevalent illness.

## Materials and methods

### Study design, setting, and population studied

A cross-sectional epidemiological study was carried out in July 2017 on residents 18 years old and older in Tsarang village, Dhaulagiri zone, Mustang district of Western Nepal. Mustang is located in Province number 4 which is one of the seven administrative divisions of Nepal. The total population of Tsarang was reported 452 persons (M:F = 217:235) in 132 households. The number of males and females above the age of 18 years are 179 and 190 respectively [11]. The migration rates in Province number 4 are 27% and 19.2% for men and women respectively [17]. Considering the migration rates, total eligible participants in the study were 285. Assuming 95% confidence interval, 5% level of significance, and 50% prevalence of NCDs, the total estimated sample size was 190. Tsarang villagers were notified in advance of the small medical clinic we had set up for the survey. From those who visited the clinic, we recruited 188 participants (M:F = 85:103) between the age of 18 and 80 years that gave a response rate of above 65%. A random sampling method was used to select the study participants from the eligible population.

The questionnaire to investigate on the lifestyle, socioeconomic status, and demographic data was a modified version of the WHO Stepwise approach to surveillance (STEPs) [18]. Each participant was interviewed by a trained local nurse. We set inclusion and exclusion criteria as follows: inclusion criteria on individuals ≥ 18 years old, who had been living at a high altitude/that research site (≥ 3500 m), and at present, been there for at least 3 months continuously. Exclusion criteria were pregnant women, residents with severe illness such as cancer, those refusing consent, those who are not from that mountain region, or those who have been there for less than 3 months.

### Biochemical and physical examinations

Trained health care providers took anthropometric measurements for height, weight, WC, and hip circumference. Other measurements included hemoglobin (Hb) concentration in blood, heart rate, oxygen saturation, blood pressure (BP), and glycosylated hemoglobin (HbA1c) and were taken by medically trained staffs. WC and hip circumference were measured with subjects standing and breathing normally. BMI was defined as the body mass (kg)/height^2^ (m^2^). BP and heart rate were measured by a digital automatic BP monitor (OMRON Model, HEM-7210, Kyoto, Japan), and an average of the two readings was recorded as participant’s BP. Systolic BP (SBP) ≥ 140 mmHg and/or diastolic BP (DBP) ≥ 90 mmHg or self-report physician diagnosis are categorized as hypertensive [19].

To measure the level of HbA1c, blood samples were analyzed by Siemens DCA Vantage analyzer (Siemens Healthcare Diagnostics, Munich, Germany) according to the manufacturer’s instructions, and the level of HbA1c was based on the National Glycohemoglobin Standardization Program (NGSP). HbA1c levels between 6 and < 6.5% are defined as intermediate hyperglycemia (IHG) or pre-DM, and at HbA1c level ≥ 6.5% as DM [20]. Hb concentration was measured by ASTRIM FIT health monitoring analyzer (Sysmex, Kobe, Japan) according to the instructions. Hb concentrations were classified into different groups as anemia (Hb < 13 g/dl for males and < 12 g/dl for females), normal (Hb ≥ 13 g/dl to < 18 g/dl for males, Hb ≥ 12 g/dl to < 16 g/dl for females), and polycythemia (≥ 18 g/dl for males, ≥ 16 g/dl for females) respectively. Oxygen saturation of Hb (SpO_2_) was measured by a pulse oximeter (Masimo Radical V 5.0, Masimo Corp, CA, USA). When SpO_2_ ≥ 90%, it was defined as normoxia. When SpO_2_ < 90%, it was defined as hypoxemia. Overweight and obesity combined population was defined as BMI ≥ 25 [21]. Central obesity was defined based on WC (≥ 90 cm for male and ≥ 80 cm for female) and waist-to-hip ratio (WHR; > 0.9 for male and > 0.85 for female) [22].

### Other study variables

To understand the socioeconomic status of participants, we investigated poverty status, education, primary occupation, and marital status. To verify poverty status, we used two different parameters, multidimensional poverty index (MPI) and income poor. MPI developed by United Nations Development Programme (UNDP) is based on identification of multiple deprivations at the household level in education, health, and standard of living and is calculated by an instruction manual [23]. Income poor is defined by the World Bank and set at 1.90 USD/day as the international poverty line (1 USD = 103 NPR). In addition, we also asked participants about family and previous histories to understand about major health concerns in participants, and about participant’s behavior towards consumption of tobacco, fruits and vegetables, and alcohol as illustrated by the WHO STEPs questionnaire.

### Statistical analysis

Data were analyzed by *χ*^2^ test and Fisher’s exact test for comparison of the rates of variables and by the Welch *t* test and Mann-Whitney U test for comparison of variables between males and females. The 95% confidential interval (CI) for a single proportion was calculated by the Clopper-Pearson method. In our analyses, results represented as mean ± standard deviation (SD) were calculated using continuous variables, while results indicated as proportions (%) with 95% CI were using stratified variables. We conducted analyses for trend across the age groups of values to verify the statistically significant differences in each of research variables. Multiple logistic regression analysis was conducted to evaluate the relationship between the risk for the prevalence of IHG and DM combined population (IHG/DM) as a dependent variable and each of independent variables with expression by odds ratio (OR) with 95% CI by a backward stepwise procedure. Age, BMI, WC, SBP, DBP, Hb concentration, MPI poor index, and daily income were used as continuous independent variables, whereas sex, SpO_2_, education, primary occupation, and marital status were as categorical independent variables respectively. Akaike’s information criterion (AIC) was used for comparison of logistic regression models. Tests results with *p* values < 0.05 were considered to be statistically significant. All statistical analyses were performed using Software R (version 3.4.1).

## Results

### Demographic, lifestyle, and socioeconomic characterizations of participants

A total of 188 participants, 85 (45.2%) were males and 103 (54.8%) were females between 18 and 80 years old participated in this study (Table 1). Some participants did not measure the values of HbA1c (*n* = 14) and Hb concentration (*n* = 10) due to instrument failures and severe swelling or deformity of finger joints.

**Table 1:** Demographic and lifestyle characterizations of participants by gender

Variables	All	Male	Female	*P* value
Sex (*n*)	188	85 (45.21)	103 (54.79)	
Age (mean ± SD)	46.31 ± 14.66	45.74 ± 13.40	46.79 ± 15.67	0.6694
Age (median; IQR)	45 (22)	43 (20)	47 (25)	
Marital status (%)^a^				
Currently married	62.77 (55.39–69.61)	75.29 (64.54–83.73)	52.43 (42.40–62.27)	0.0017
Not currently married	36.17 (29.39–43.52)	24.71 (16.27–35.46)	45.63 (35.88–55.71)	
Education (%)				
No schooling	68.09 (60.84–74.58)	47.06 (36.25–58.13)	85.44 (76.80–91.35)	< 0.0001
Less than primary school completed	6.91 (3.89–11.79)	11.76 (6.09–21.01)	2.91 (0.76–8.90)	
Less than secondary school completed	12.23 (8.07–17.99)	20.00 (12.40–30.36)	5.83 (2.39–12.75)	
More higher education	12.77 (8.51–18.59)	21.18 (13.36–31.65)	5.83 (2.39–12.75)	
Primary occupation (%)				
Employed	26.60 (20.55–33.61)	43.53 (32.95–54.70)	12.62 (7.16–20.97)	< 0.0001
Unemployed	73.40 (66.39–79.45)	56.47 (45.30–67.05)	87.38 (79.03–92.84)	
Smoking habits (%)				
Current/Ex-smoker	21.28 (15.80–27.96)	34.12 (24.40–45.29)	10.68 (5.71–18.69)	0.0002
Non smoker	78.72 (72.04–84.20)	65.88 (54.71–75.60)	89.32 (81.31–94.29)	
Alcohol drinking habits (%)^b^				
Current/Ex-drinker	32.09 (25.57–39.35)	50.00 (39.54–60.46)	17.48 (10.96–26.48)	< 0.0001
Non drinker	67.91 (60.65–74.43)	50.00 (39.54–60.46)	82.52 (73.52–89.04)	
Vegetable consumption (in a week; %)^b^				
Less than once	14.44 (9.89–20.49)	19.05 (11.60–29.38)	10.68 (5.71–18.69)	0.2123
2–3 days	65.24 (57.90–71.95)	59.52 (48.24–69.93)	69.90 (59.95–78.34)	
More than 4 days	20.32 (14.94–26.95)	21.43 (13.52–31.40)	19.42 (12.54–28.63)	
Vegetable and fruit serving (in a day; %)^b^				
Less than one	18.18 (13.08–24.62)	20.24 (12.56–30.70)	16.50 (10.18–25.40)	0.2385
2–3 servings	68.98 (61.75–75.42)	63.10 (51.81–73.17)	73.79 (64.03–81.73)	
More than 4 servings	12.83 (8.56–18.69)	16.67 (9.73–26.73)	9.71 (5.01–17.54)	

Values are in percentage (95% CI). Data were analyzed by *χ*^2^ test or Fisher’s exact test for comparison of the rates of variables and by the Mann-Whitney U-test for comparison of variables between males and females

*SD* standard deviation, *IQR* interquartile range, *CI* confidential interval

^a^Two participants refused to answer

^b^One participant refused to answer

Most of participants (68.1%) had no formal education or did not finish primary school, and females (85.4%) had almost two-fold higher proportion of no formal education than males (47.1%). Higher proportions of males than females were employed (43.5% vs 12.6%) and had smoking and alcohol drinking habits (34.1% and 50.0% vs 10.7% and 17.5%). When we asked dietary habits of participants about fresh vegetable and fruits consumption, nearly 70% of participants said they consumed them around 2–3 days in a week (Table 1). On economic status, 71% of participants reported ≤ 100,000 NPR as expected annual household income (Table 2). We used two parameters; MPI poor and income poor, which is relatively a subjective and objective indexes of poor respectively, to evaluate their economic conditions. The national levels of MPI poor and income poor in Nepal are 28.6% and 14.9%, respectively [24]. As shown in Table 2, our study showed that about 27% of the participants were MPI poor, whereas almost half of the participants were as income poor, indicating that the proportion of income poor is higher than the national status of Nepal. These results indicate that people living at Tsarang have established lifestyles without being dependent on cash income and achieved a standard of living equivalent to a nationwide. By self-reports about family and previous histories, many participants complained of joint pain, indicating that arthritis or osteoarthritis might be a major health concerns in participants (Table 2).

**Table 2:** Socioeconomic characterization and disease history of participants

Factors	*n* (%)
Expected annual income (NPR)^a^	
≤ 10,000	22 (12.29)
10,001 to ≤ 50,000	43 (24.02)
50,001 to ≤ 100,000	62 (34.64)
> 100,000	52 (29.05)
Poverty	
MPI poor^b^	51 (27.27)
Income poor^a^	90 (50.28)
Family history^c^	
Arthritis	74 (41.11)
Hypertension	18 (10.00)
Rheumatism	11 (6.11)
Others^d^	6 (3.33)
Previous history^c^	
Hypertension (or raised blood pressure)	32 (17.02)
Diabetes (or raised blood sugar)	6 (3.19)
Allergy	60 (32.26)
Osteoarthritis^e^	116 (62.70)

^a^9 participants are excluded because they do not know their own annual income

^b^MPI poor is defined as deprivation score ≥ 33.3% [23]. *MPI*, multidimensional poverty index

^c^Self-reported by participants

^d^Including allergy, diabetes, and obesity

^e^Including joint pain, swollen joints, and stiff joints

### Anthropometric and biochemical characteristics by sex and age

BMI, WC, and WHR were calculated to find the prevalence of overweight, obesity, and central obesity respectively. The combination of being overweight and obese was observed in 29.3% of participants with no significant difference between sexes (Table 3). Central obesity calculated by WHR had higher prevalence in females than males with rates of 59.4% and 40.2% respectively, whereas by WC, it had 23.5% in total, with females having higher prevalence of 31.1% than males who had it 14.3%. 27.1% for males versus 15.5% for females were hypertensive. A total of 20.7% of participants were hypertensive and most had normal Hb concentration. To examine the prevalence of type 2 DM in participants, we measured HbA1c because this value is considered to be a good risk marker for the development of type 2 DM [25]. In this study, we found that 31.6% and 4.6% of participants were classified as IHG and DM respectively (Table 3). The prevalence of DM as well as IHG increased steadily as the age advanced. Among the age group ≤ 40 years, almost all participants had normal range of HbA1c, whereas the prevalence of IHG/DM rose dramatically for those over 40 years of age (Table 4).

**Table 3:** Anthropometric and biochemical characteristics of studied variables by sex

Variables	Total	Male	Female	*P* value
Height (cm)	157.11 ± 9.30	164.18 ± 6.81	151.27 ± 6.66	< 0.0001
Weight (kg)	57.59 ± 10.44	62.59 ± 9.39	53.46 ± 9.45	< 0.0001
BMI	23.28 ± 3.43	23.21 ± 3.17	23.34 ± 3.64	0.8780
Overweight (BMI ≥ 25) (%)	29.26 (22.98–36.40)	30.59 (21.30–41.66)	28.16 (19.95–38.01)	0.8384
WC (cm)	77.27 ± 8.68	79.43 ± 8.42	75.50 ± 8.52	0.0017
≥ 90 cm (male)/≥ 80 cm (female) (%)	23.53 (17.78–30.39)	14.29 (7.92–24.02)	31.07 (22.52–41.05)	0.0118
Hip (cm)^a^	88.86 ± 7.92	90.00 ± 7.23	87.94 ± 8.36	0.0499
WHR^a^	0.87 ± 0.05	0.88 ± 0.06	0.86 ± 0.05	0.0062
> 0.9 (male)/> 0.85 (female) (%)	50.82 (43.37–58.24)	40.24 (29.74–51.67)	59.41 (49.16–68.93)	0.0151
SpO_2_ (%)	90.60 ± 3.21	90.47 ± 3.48	90.71 ± 2.97	0.9913
Hypoxemia (SpO_2_ < 90%) (%)	27.13 (21.04–34.17)	28.24 (19.26–39.20)	26.21 (18.27–35.97)	0.8843
Heart rate (bpm)	81.57 ± 12.13	81.35 ± 13.74	81.76 ± 10.68	0.8249
SBP (mmHg)	122.67 ± 18.22	128.36 ± 16.77	117.97 ± 18.11	< 0.0001
≥ 140 mmHg (%)	17.02 (12.09–23.33)	23.53 (15.29–34.20)	11.65 (6.43–19.84)	0.0498
DBP (mmHg)	77.07 ± 11.61	79.67 ± 10.72	74.92 ± 11.92	0.0030
≥ 90 mmHg (%)	13.30 (8.95–19.19)	16.47 (9.62–26.43)	10.68 (5.72–18.69)	0.3431
HT^b^	20.74 (15.33–27.39)	27.06 (18.26–37.96)	15.53 (9.41–24.30)	0.0786
Hb (g/dL)^c^	13.82 ± 1.74	14.48 ± 1.42	13.33 ± 1.80	< 0.0001
< 13 (male)/< 12 (female) (%)	17.42 (12.31–23.97)	14.47 (7.79–24.85)	19.61 (12.66–28.89)	0.0028
13 to < 18 (male)/12 to < 16 (female) (%)	76.40 (69.35–82.29)	85.53 (75.15–92.91)	69.61 (59.59–78.12)	
≥ 18 (male)/≥ 16 (female) (%)	6.18 (3.28–11.07)	0	10.78 (5.77–18.87)	
HbA1c (NGSP; %)^d^	5.96 ± 0.99	5.90 ± 1.00	6.01 ± 0.98	0.1051
6.0 to < 6.5% (%)	31.61 (24.90–39.15)	27.27 (18.04–38.81)	35.05 (25.83–45.48)	0.5544
≥ 6.5% (%)	4.60 (2.15–9.17)	5.19 (1.68–13.47)	4.12 (1.33–10.82)	

Values are mean ± SD and percentage (95% CI). Data were analyzed by *χ*^2^ test or Fisher’s exact test for comparison of the rates of variables and by the Welch t-test or Mann-Whitney U-test for comparison of variables between males and females

*BMI* body mass index, *WC* waist circumference, *WHR* waist-to-hip ratio, *SBP* systolic blood pressure, *DBP* diastolic blood pressure, *HT* hypertension, *Hb* hemoglobin, *SD* standard deviation, *CI* confidential interval

^a^5 participants refused measurement

^b^≥ 140 mmHg SBP and/or ≥ 90 mmHg DBP in participants

^c^10 participants could not be measured due to severe deformation of finger joint or machine troubles

^d^14 participants could not be measured due to machine troubles

**Table 4:** Prevalence of IHG/DM in sex by age groups

	Male (*n* = 77)			Female (*n* = 97)		
	HbA1c < 6.0%	HbA1c ≥ 6.0%	*P* for trend	HbA1c < 6.0%	HbA1c ≥ 6.0%	*P* for trend
Age ≤ 30	6	1	0.0002	16	1	< 0.0001
31 to ≥ 40	22	1		16	3	
41 to ≥ 50	13	8		12	9	
51 to ≥ 60	7	9		11	9	
> 60	4	6		4	16	

Data were analyzed by Cochran-Armitage trend test for comparison of prevalence of participants. *IHG/DM*, intermediate hyperglycemia and diabetes mellitus combined population

### Factors that affect participants’ health

To estimate the association between the risk for IHG/DM in participants and research variables (sex, age, BMI, WC, SpO_2_, SBP, DBP, Hb concentration, MPI index, daily income, education, marital status, primary occupation), we first conducted univariate logistic regression analyses of each of variables (Table 5). In the univariate regression analyses, the prevalence of IHG/DM was positively associated with age (*p* < 0.0001, for every 1 year increase) and Hb concentration (*p* = 0.0339, for every one unit increase). In addition, participants with hypoxemia (*p* < 0.0001) were at the increased risk for the prevalence of IHG/DM than persons with normoxia. We next performed an analysis to identify the association between the prevalence of IHG/DM and multivariate confounding factors by a stepwise multiple logistic regression analysis. On this analysis, age, BMI, SpO_2_, SBP, Hb concentration, and marital status remained as confounders, and therefore we conducted further calculation of ORs for the prevalence of IHG/DM using those remaining confounders as independent variables. As shown in Table 5, age (OR = 1.113, 95% CI = 1.063–1.165, *p* < 0.0001, for every 1 year increase) and BMI (OR = 1.260, 95% CI = 1.081–1.468, *p* = 0.0031, for every one point increase) were found to be positively associated with the prevalence of IHG/DM. Residents with hypoxemia were at the increased prevalence for the IGH/DM compared to participants with normoxia (OR = 3.577, 95% CI = 1.198–10.678, *p* = 0.0224, vs normoxia). In addition, unmarried participants had a significantly increased prevalence for IHG/DM than married participants (OR = 2.717, 95% CI = 1.001–7.374, *p* = 0.0498, vs currently married participants).

**Table 5:** Stepwise logistic regression analyses to evaluate relationships between research variables and the prevalence of IHG/DM

Variables	Crude			Adjusted		
	OR	95% CI	*P* value	OR	95% CI	*P* value
Age	1.1111	1.0736–1.1562	< 0.0001	1.1128	1.0631–1.1647	< 0.0001
BMI	1.0537	0.9499–1.1703	0.3220	1.2596	1.0811–1.4675	0.0031
SpO_2_						
≥ 90%	Ref			Ref		
< 90% (hypoxemia)	7.9222	3.4488–19.3937	< 0.0001	3.5769	1.1983–10.6775	0.0224
Hb concentration	1.2370	1.0200–1.5150	0.0339	1.3093	0.9845–1.7412	0.0634
SBP	0.9999	0.9813–1.0182	0.9920	0.9778	0.9532–1.0030	0.0832
Marital status						
Currently	Ref			Ref		
Not currently	1.9397	0.9513–3.9548	0.0673	2.7168	1.0009–7.3741	0.0498
Sex						
Male	Ref					
Female	1.6941	0.8479–3.4699	0.1408			
WC	1.0343	0.9952–1.0761	0.0892			
DBP	0.9956	0.9667–1.0247	0.7670			
MPI poor index	1.1112	0.0753–15.2549	0.9374			
Daily income	0.9608	0.8656–1.0524	0.4145			
Education						
Less than primary school completed	Ref					
Less than secondary school completed	0.4286	0.1169–1.2598	0.1522			
More higher education	0.4571	0.1241–1.3564	0.1885			
Primary occupation						
Employed	Ref					
Unemployed	1.7222	0.7855–4.0305	0.1888			
AIC		–			140.13	

Univariate logistic regression analyses were conducted to the association between the prevalence of IHG/DM and each of variables by expression of OR with 95% CI (left). After conduction of a stepwise multiple logistic regression analysis using all research variables, ORs were calculated using remained confounders by a multiple logistic regression analysis (right)

*IHG/DM* intermediate hyperglycemia and diabetes mellitus combined population, *OR* odds ratio, *CI* confidential interval, *BMI* body mass index, *Hb* hemoglobin, *SBP* systolic blood pressure, *WC* waist circumference, *DBP* diastolic blood pressure, *MPI* multidimensional poverty index, *AIC* Akaike’s Information Criterion, *DM* diabetes mellitus

## Discussion

The present study is an epidemiological investigation of rural highlanders of Mustang district, Nepal, that included information on demography, socioeconomic and current health condition. As shown in previous reports, the prevalence of NCDs in Nepal is increasing in recent years, and therefore NCDs are recognized as an emerging health concern [6, 26]. To our knowledge, epidemiological information on NCD prevalence in the Mustang district is limited, and it is difficult to accurately assess the health status of isolated villages.

Our study showed the prevalence of overweight and obese participants to be 29.3% (95% CI = 23.0–36.4%). A study conducted at other villages in Mustang district, Nepal, reported the prevalence of overweight and obese population at the rural setting to be 26.7% whereas at the urban settings to be 47.7% and 56.6% respectively [27]. A possible reason behind these differences may be associated with economic status. In addition, a nationwide survey in Nepal done in 2013 showed the prevalence of overweight and obese population that inhabitants of the mountain regions to be 9.0% (95% CI = 4.6–17.0%) [6]. These differences between Tibetans in Mustang district and nationwide survey could be attributed to be Tibetans’ increased preference for high consumption of carbohydrates and physical inactivity coined with low education level of the population studied [28]. The prevalence of pre-DM or IHG in our study participants was 31.6% (95% CI = 24.9–39.2%) (Table 3). Our result is similar as in other findings examining the prevalence of IHG in different villages that have the same ethnic background as in our study. For instance, research at other villages in Mustang district has observed the prevalence of IHG ranging from 22.1–39.3% [27]. In addition, examining of the prevalence of IHG at the same altitude in China and India ranged from 22.3–41.6% [29, 30]. On the other hand, a meta-analysis of data that examined the prevalence of DM and IHG in Nepal found pre-DM population to be 10.3% (95% CI = 6.1–14.4%) [10]. The study population for this meta-analysis included various ethnic groups not restricted to a specific profession. Therefore, these observations indicate that ethnicity, lifestyle habits, and socio-economic characteristics may attribute to these observed differences.

On a stepwise multiple logistic regression analysis, we found that lifestyle and health-related factors were significantly associated with the prevalence of IHG/DM in our participants (Table 5). Note that, participants with hypoxemia had increased prevalence of IHG/DM compared with participants with normoxia. A previous study has shown that hypoxemia plays a role in the high prevalence of glucose intolerance in Tibetan people of India and China [30]. Although, regarding the association between hypoxemia and glucose intolerance or elevation of HbA1c levels, several reports related to patients with sleep disorder could find that there is significant correlation between HbA1c or blood glucose level and SpO_2_, associated mechanisms of hypoxemia with glucose intolerance are not clearly known [31, 32]. We speculate a possible mechanism that hypoxic environment generates both hypoxemia and oxidative stress, causing induction of expression of many genes including that related to inflammatory pathways, resulting in hyperglycemia [33–35]. In addition, both elevation of HbA1c value and the prevalence of hypoxemia are accelerated by aging process [30, 36, 37]. When we examined the correlation of age with SpO_2_ or HbA1c in participants by Spearman rank correlation tests, SpO_2_ (*ρ* = − 0.535, *p* < 0.0001) was negatively associated with age, whereas HbA1c (*ρ* = 0.554, *p* < 0.0001) was positively, indicating that both prevalence of hypoxemia and IHG are dependent on aging. Therefore, we speculate that aging process causes hypoxemia and glucose intolerance, despite normal Hb concentration among elderly participants (Table 6). In the present study, the prevalence of IHG was remarkably high (31.6%), particularly among the participants above the age of 40 years. This suggests that there will be an increased burden of DM at the study site as the population ages. The challenge for future research efforts should focus on prevention and control measures for NCDs in rural highlands in areas with limited access. Early detection, screening, and treatment of NCDs, as well as palliative care, are key components of the response to NCDs which present a serious challenge to resources in developing countries such as Nepal. Hence, sustainable and effective measures for detection, screening, and treatment of NCDs targeting the rural highlanders in Nepal need to be urgently developed. The results from our study may contribute to the limited body of research which we hope will lead to effective measures for detection, screening, and treatments of NCDs.

**Table 6:** Tendency and prevalence of variables in sex by age groups

Sex	Variables	Age ≤ 40	41 to ≤ 60	> 60	*P* for trend
Male	Hb (g/dL)^a^	14.72 ± 1.44	14.51 ± 1.38	13.65 ± 1.31	0.0328
	SpO_2_ (%)^a^	92.27 ± 1.59	89.53 ± 3.65	88.79 ± 4.51	< 0.0001
	IHG/DM (%)^b^	6.67 (1.16–23.51)	45.95 (29.85–62.87)	60.00 (27.37–86.31)	0.0001
Female	Hb (g/dL)^a^	12.61 ± 1.69	13.59 ± 1.62	14.05 ± 1.91	0.0004
	SpO_2_ (%)^a^	92.39 ± 1.67	90.62 ± 2.87	88.09 ± 2.97	< 0.0001
	IHG/DM (%)^b^	11.11 (3.62–27.00)	43.90 (28.82–60.11)	80.00 (55.73–93.39)	< 0.0001

*Hb* hemoglobin, *IHG/DM* intermediate hyperglycemia and diabetes mellitus combined population

^a^Values are mean ± SD. Data were analyzed by Jonckheere-Terpstra trend test for comparison of means of variables among age. SD, standard deviation

^b^Values are represented as proportion of prevalence of IHG/DM (95% CI). Data were analyzed by Cochran-Armitage trend test for comparison of proportion of IHG/DM among age groups. *CI*, confidential interval

In this study, we found that hypoxemia, age, BMI, and marital status are the factors for the high prevalence of IHG/DM (Table 5). Previous studies have shown that many Tibetan residents living at high altitude have low oxygen saturation levels [38, 39]. Therefore, we speculate that hypoxemia is one of the common factors for the high prevalence of IHG/DM in Tibetan highlanders because our finding is in accordance with previous reports from different countries of the same ethnic population from different countries [30]. Further research in other high-altitude communities as well as lowland residents with the same ethnic background may confirm the risk factors that our study has revealed, and it may lead to establishing appropriate prevention and control strategies of cardio-metabolic diseases in the highland areas of Nepal. In addition, it has been recently shown that genetic polymorphisms in the gene encoding EGLN1 and in mitochondrial-DNA (mtDNA) are associated with SpO_2_ responses [40, 41]. However, these studies were conducted in lowland Japanese cohort and in the acute hypobaric hypoxia. So, it might be difficult to discuss this association because our study population is different ethnic groups and highland residents. But, such genetic studies will provide the new insight into the effects in physiological responses of our study population.

It should be noted that this study has several limitations. The first is the involvement of only one research site which was a result of difficult access. However, this study has encouraged us to confirm our findings in other areas and follow up studies. Second, we measured HbA1c as a diagnostic test for IHG and DM. This survey did not measure fasting blood samples which could have given the definitive diagnosis. However, HbA1c requires no patient preparation, e.g., fasting before blood collection, which could be difficult because of participants’ education levels and cultural views. The present study has shed light on our limitation and encouraged us for our next research plan to evaluate the associations between genetic polymorphisms of our population and their physiology of hypoxemia or the disease processes.

## Conclusions

Our results suggest that there is an increased prevalence of IHG/DM for hypoxic people of 40 years of age and above in Tsarang village of rural Nepal suggesting an urgent need for prioritizing public health concern for NCDs. The association of hypoxemia and prevalence of IHG/DM as shown by this study could be due to the additive effects of hypoxia, lifestyle or dietary habits, and genetic predisposition of the Tibetan highlanders. More population-specific studies are the required to aim for estimation of prevalence, prevent, and control NCDs in the mountainous regions.

## Abbreviations

AIC: Akaike’s information criteria
BMI: Body mass index
BP: Blood pressure
CI: Confidence interval
DBP: Diastolic blood pressure
DM: Diabetes mellitus
Hb: Hemoglobin
HbA1c: Glycosylated hemoglobin
HT: Hypertension
IHG: Intermediate hyperglycemia
MPI: Multidimensional poverty index
NCDs: Noncommunicable diseases
OPHI: Oxford poverty and human development initiative
OR: Odds ratio
SBP: Systolic blood pressure
SpO_2_: Oxygen saturation of hemoglobin
STEPs: Stepwise approach to surveillance
UNDP: United Nations Development Programme
WC: Waist circumference
WHO: World Health Organization
WHR: Waist-to-hip ratio
